# Application of Hydrotropic Solubilization in Spectrophotometric Estimation of Lornoxicam from Tablets

**DOI:** 10.1155/2014/810128

**Published:** 2014-10-29

**Authors:** Sindhu Abraham, Rajamanickam Deveswaran, Sharon Furtado, Srinivasan Bharath, Varadharajan Madhavan

**Affiliations:** ^1^Department of Pharmaceutics, M.S. Ramaiah College of Pharmacy, MSRIT Post, MSR Nagar, Bangalore 560054, India; ^2^Department of Pharmacognosy, M.S. Ramaiah College of Pharmacy, MSRIT Post, MSR Nagar, Bangalore 560054, India

## Abstract

Lornoxicam is a selective cyclooxygenase-1 and cyclooxygenase-2 inhibitor that exhibits anti-inflammatory, analgesic, and antipyretic activities. It is used in osteoarthritis and rheumatoid arthritis; and in treatment of postoperative pain and primary dysmenorrhoea. Lornoxicam is completely insoluble in water but soluble in alkaline solutions. Hydrotropic solubilization is a technique used to increase the aqueous solubility of poorly water-soluble drugs and the present study was aimed at developing a hydrotropic technique to increase the solubility of lornoxicam, using 2 M sodium benzoate as the hydrotropic agent. Beer's law was obeyed in the concentration range of 4–24 *μ*g/mL at 381 nm. The solubility of lornoxicam in distilled water considerably increased with the addition of a hydrotropic agent. The analysis of tablets indicated good correlation between the amounts estimated and label claim. The LOD and LOQ of lornoxicam were found to be 0.34 *μ*g/mL and 1.038 *μ*g/mL, respectively, indicating good sensitivity of the proposed method. The percentage recovery was found to be 99.99%–100.21%. Thus the proposed method is new, simple, environmentally friendly, accurate, and cost effective and can be successfully employed in routine analysis of lornoxicam in tablets.

## 1. Introduction

Various techniques have been employed to enhance the aqueous solubility of poorly water-soluble drugs. Hydrotropic solubilization is one such method [1]. Hydrotropes are a class of chemical compounds which affect an increased aqueous solubility by several folds to certain solutes which are sparingly soluble in water under normal conditions. Therapeutic efficacy of a drug depends upon its bioavailability and ultimately its solubility to achieve a desired concentration in systemic circulation. Because of their low aqueous solubility and high permeability, dissolution from delivery systems forms the rate limiting step in their absorption and systemic bioavailability [2, 3]. Increasing the aqueous solubility of insoluble and slightly soluble drugs is of major importance and hydrotropy can be considered as a potential and industrially attractive technique because of easy recovery of dissolved solute and possible reuse of hydrotrope solutions. Hydrotropes such as sodium benzoate, sodium salicylate, niacinamide, sodium hydroxide, sodium citrate, PEG 6000, polyvinyl alcohol, and urea have been employed to enhance the aqueous solubility of poorly water-soluble drugs [3].

Lornoxicam, an oxicam derivative, is a nonsteroidal anti-inflammatory drug, is a selective cyclooxygenase-1 and cyclooxygenase-2 (COX-1 and COX-2) inhibitor, and exhibits anti-inflammatory, analgesic, and antipyretic activities. It is used in muscular, skeletal, and joint disorders such as osteoarthritis and rheumatoid arthritis. It is also used in the treatment of other painful conditions including postoperative pain and primary dysmenorrhoea.

Lornoxicam is practically insoluble in water but soluble in alkaline solutions. Most of the previous methods employed have used organic solvents or hydrotropes such as sodium lauryl sulphate and urea to increase aqueous solubility of lornoxicam [4, 5]. In the present study, a new, simple, accurate, cost effective, and sensitive spectrophotometric method was developed for the estimation of lornoxicam in tablets using 2 M sodium benzoate as a hydrotropic agent. This method could preclude the use of costly solvents in analysis.

## 2. Materials and Methods

### 2.1. Materials

UV/visible spectrophotometer (Model-UV-1700, Shimadzu, Japan) was employed for the spectral measurements. Lornoxicam was a generous gift sample from Life Care Formulations Pvt. Ltd., Pondicherry. Commercial tablets of lornoxicam (LORSAID 4, Abbott Healthcare Pvt. Ltd., Mumbai) were purchased from the local pharmacy. All other chemicals and solvents used were of analytical grade.

### 2.2. Preliminary Solubility Studies of the Drug

Solubility of lornoxicam was determined by saturation aqueous solubility method in 2 M sodium benzoate. An excess amount of drug was added to the 100 mL beakers containing mixture of 2 M sodium benzoate. The beakers were shaken for 12 hours at 28 ± 1°C. The solutions were filtered through Whatman filter paper No. 41, and the resulting filtrates were suitably diluted and analyzed spectrophotometrically at 381 nm against solvent blank [6].

### 2.3. Preparation of Standard Stock and Calibration Curve

**Figure 1:**
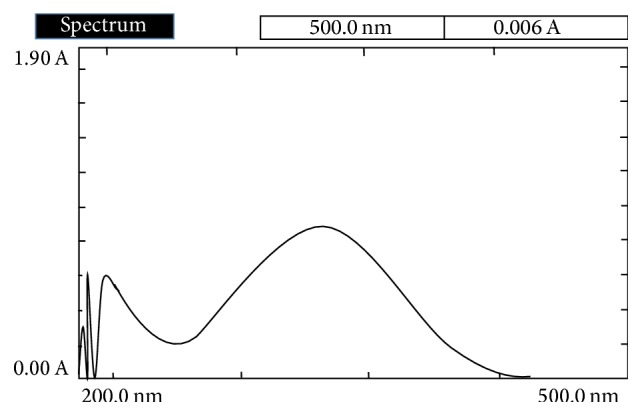
UV spectrum of lornoxicam in 2 M sodium benzoate as hydrotropic agent.

The standard stock solution of lornoxicam was prepared by dissolving 50 mg of drug in 50 mL of 2 M sodium benzoate. From this stock solution 5 mL was diluted to 50 mL with distilled water to get a concentration of 100 *μ*g/mL and scanned in the entire UV range of 400–200 nm to determine the *λ* max of the drug. The *λ* max of lornoxicam was found to be 381 nm (Figure 1). Six working standard solutions for the drug having concentrations 4, 8, 12, 16, 20, and 24 *μ*g/mL were prepared with distilled water from the stock solution. The absorbances of the resulting solutions for the drug were measured at 381 nm and a calibration curve was plotted to get the linearity and regression equation.

### 2.4. Linearity

From the stock solution, serial dilutions were made and the absorbances of solutions were measured at the respective wavelength as per the developed method to confirm the linearity.

### 2.5. Validation of the Proposed Method

#### 2.5.1. Recovery Studies

In order to check the accuracy and reproducibility of the proposed method, recovery studies were conducted. Tablet powder (commercial formulation) equivalent to 4 mg of lornoxicam was transferred to a 50 mL volumetric flask containing 40 mL of 2 M sodium benzoate. Pure lornoxicam sample (2 mg) was added to the same volumetric flask. The flask was shaken for 5 min to solubilize the drug. The solution was then filtered through Whatman filter paper No. 41. The filtrate was diluted with distilled water appropriately and absorbance was measured at 381 nm against corresponding reagent blank. Drug content was estimated and percent recovery was calculated using the following formula. Similar procedure was repeated using 4 mg and 6 mg of pure lornoxicam as spiked concentration. Consider

(1) $$\begin{array}{c} \%\text{Recovery}=\frac{b-c}{a}\times 100, \end{array}$$

where *a* is the amount of drug found before addition of pure drug, *b* is the amount of drug found after addition of pure drug, and *c* is the amount of pure drug added.

#### 2.5.2. Precision

Precision was determined by studying the repeatability and intermediate precision. The standard deviation, coefficient of variance (CV), and standard error were calculated for the drug.

#### 2.5.3. Interday and Intraday Precision

The intraday concentration of the drug was calculated on the same day at an interval of one hour, whereas the interday concentration of drug was calculated on three different days, within the laboratory conditions [7].

#### 2.5.4. Intersubject Precision

The intersubject variation was calculated by taking into account the analysis carried out by three different individuals on the same day and at the same time, within the laboratory conditions [8, 9].

#### 2.5.5. Limit of Detection (LOD) and Limit of Quantitation (LOQ)

The LOD and LOQ of lornoxicam by the proposed method were determined using calibration standards. LOD and LOQ were calculated as 3.3*σ*/*S* and 10*σ*/*S*, respectively, where *S* is the slope of the calibration curve and *σ* is the standard deviation of response [10].

## 3. Results and Discussion

### 3.1. Preliminary Solubility Studies of the Drug

The results of solubility studies indicated that aqueous solubility of lornoxicam was enhanced in hydrotropic mixture solution of 2 M sodium benzoate as compared to solubility in distilled water. The solubility of pure lornoxicam in distilled water was found to be 0.012 mg/mL, whereas in 2 M sodium benzoate the solubility was found to be approximately 5 mg/mL. The increase in solubility was more than 100-fold. Hence this method was optimized and employed in the analysis of the tablet formulation. A portion of the solution was kept at room temperature for 24 hours to check the stability of drug in presence of sodium benzoate.

The study revealed that estimations of lornoxicam can be done within 24 hours without any detrimental effect on drug stability as no precipitation was observed. From this study it is obvious that there was no interference of 2 M sodium benzoate in estimation of lornoxicam at the wavelength of 381 nm. Based on this a large number of poorly water-soluble drugs having *λ* max above 250 nm may be tried for estimation by the proposed method provided their preliminary solubility studies confirm the enhancement of solubility in 2 M sodium benzoate. Sodium benzoate is cheaper than most of the organic solvents and thus may be a better substitute for expensive organic solvents that are used in routine analysis of pharmaceuticals.

### 3.2. Standard Stock and Calibration Curve

The Beer-Lambert's concentration range was found to be 4–24 *μ*g/mL for lornoxicam at the wavelength of 381 nm. The drug showed good regression value at this wavelength. It was evident that there is good correlation between the amounts estimated and the label claim. The estimated label claim was found to be 100.03 ± 0.217 mg with low values of standard error (Table 1).

### 3.3. Linearity and Recovery Studies

Accuracy and reproducibility of the proposed method were further confirmed by recovery studies. The results of the study revealed that any small change in the drug concentration in the solution could be accurately determined by the proposed method (Table 2).

### 3.4. Interday, Intraday, and Intersubject Precision

Repeatability results indicated the precision under the same operating conditions over a short interval of time and interassay precision. Intermediate precision study expresses variation within laboratory conditions in different days.

In intra- and interday precision and intersubject variation study, coefficient of variation was not more than 1.0% indicating good intermediate precision.

### 3.5. Limit of Detection (LOD) and Limit of Quantitation (LOQ)

The value of LOD was 0.34 *μ*g/mL and LOQ 1.038 *μ*g/mL, respectively (Table 3). These low values of LOD and LOQ indicated good sensitivity of the proposed method.

## 4. Conclusion

Thus a new method has been developed that is precise, simple, cost effective, accurate, and safe which has been validated. This proposed method which was developed on the principle of hydrotropic solubilization concept can be well employed in routine analysis of lornoxicam in tablets.

## Figures and Tables

**Table 1:** Analysis of tablet formulations of lornoxicam.

Tablet formulation	Label claim (mg)	% label claim estimated^*^ (mean ± S.D)	Standard error
Commercial tablet I	4	100.03 ± 0.217	0.1087

^*^Average of six determinations.

**Table 2:** Result of recovery studies.

Amount of lornoxicam in tablet powder (mg)	Amount of standard drug added (mg)	% recovery estimated^*^ (mean ± S.D)	Standard error
4	2	100.17 ± 0.431	0.216
4	4	100.21 ± 0.205	0.102
4	6	99.99 ± 0.013	0.006

^*^Average of six determinations.

**Table 3:** Optical characteristics data and validation parameters.

Parameters	Values for lornoxicam
Working *λ*_max_ in 2 M sodium benzoate	381 nm
Beer's law limit	4–24 *µ*g/mL
Molar absorptivity	14.537 × 10^3^
Correlation coefficient^*^	0.9998
Intercept^*^	0.0032
Slope^*^	0.03873
LOD^*^	0.34 *µ*g/mL
LOQ^*^	1.038 *µ*g/mL
Intraday precision^*^ (CV)	0.0071
Interday precision^*^ (CV)	0.0012
Intersubject precision^*^ (CV)	0.0046

^*^Average of 6 determinations.
